# Metronomic chemotherapy with oral vinorelbine (mVNR) and capecitabine (mCAPE) in advanced HER2-negative breast cancer patients: is it a way to optimize disease control? Final results of the VICTOR-2 study

**DOI:** 10.1007/s10549-016-4009-3

**Published:** 2016-10-17

**Authors:** M. E. Cazzaniga, L. Cortesi, A. Ferzi, L. Scaltriti, F. Cicchiello, M. Ciccarese, S. Della Torre, F. Villa, M. Giordano, C. Verusio, M. Nicolini, A. R. Gambaro, L. Zanlorenzi, E. Biraghi, L. Legramandi, E. Rulli, On behalf of VICTOR Study Group, Francesca Riva, Pelizzoni Davide, Isabella Marchi, Elena Collovà, Giuseppe Prati, Antonio Ardizzoia, Davide Toniolo, Palma Pugliese, Claudia Pogliani, Abbondanza Gambino, Lucia Stocchi, Andrea Colombo, Cinzia Fasola, Raffaele Venezia, Fabio Galli, Valter Torri

**Affiliations:** 1 Oncology Unit, ASST Monza, Via Pergolesi, 33 20900 Monza, MB Italy; 2 Haematology and Oncology Unit, Azienda Ospedaliero-Universitaria Policlinico di Modena, Modena, Italy; 3 Oncology Unit, ASST Ovest Milanese, Ospedale di Legnano, Legnano, Italy; 4 Oncology Day Hospital Unit, Ospedale Civile di Guastalla, Guastalla, Italy; 5 Oncology Unit, Ospedale Vito Fazzi, Lecce, Italy; 6 Oncology Unit, ASST Rhodense-Presidio di Garbagnate Milanese e Presidio di Rho, Garbagnate, Italy; 7 Oncology Unit, ASST, Lecco, Italy; 8 Oncology Unit, ASST Lariana, Como, Italy; 9 Oncology Unit, ASST della Valle Olona, Saronno, Italy; 10 Oncology Day Hospital Unit, Azienda USL Romagna, Cattolica, Italy; 11 Oncology Unit, ASST Fatebenefratelli-Sacco, Milan, Italy; 12 Oncology Unit, ASST della Valle Olona, Busto Arsizio, Italy; 13 Oncology Unit, ASST Melegnano e Martesana, Gorgonzola, Italy; 14 Methodology for Clinical Research Laboratory, IRCCS Istituto di Ricerche Farmacologiche Mario Negri, Milan, Italy

**Keywords:** Metronomic chemotherapy, Vinorelbine, Capecitabine, Breast cancer

## Abstract

**Purpose:**

The VICTOR-1 study demonstrated that the all-oral metronomic combination of vinorelbine and capecitabine is highly active and well tolerated in hormone receptor-positive/HER2-negative patients. The VICTOR-2 study was designed to confirm these results.

**Methods:**

Patients received mVNR 40 mg three times a week and mCAPE 500 mg three times a day, continuously. The primary endpoint was the clinical benefit rate (CBR); secondary endpoints were toxicity, objective response rate (ORR), and progression-free survival (PFS).

**Results:**

Eighty patients were evaluable for the primary efficacy analysis. Median age was 65.3 years; most patients had HR-positive tumors (65 %). The CBR was 45.7 % (95 % CI 28.8–63.4) and 51.1 % (95 % CI 35.8–66.3) in first- and ≥ second-line therapy, respectively. The ORR was 35.5 % in first-line (95 % CI 19.2–54.6) and 25.6 % in ≥second-line (95 % CI 13.5–41.2). The median duration of response was 11.3 and 6.4 months and PFS rates at 1 year were 24.3 and 22.2 %, respectively. In triple-negative breast cancer patients (*N* = 28, 35 %) a lower, but clinically relevant CBR (35.7, 95 % CI 18.6–55.9) was observed. The main toxicities per cycle were non-febrile neutropenia (1.1 %), hand-foot syndrome (1.0 %), nausea and vomiting (1.0 %), leucopenia (0.8 %), fatigue (0.7 %), and diarrhea (0.4 %).

**Conclusion:**

The VICTOR-2 study confirms the clinical activity of mVNR and mCAPE in HER2-negative breast cancer patients, suggesting that the easy schedule of administration, which requires monthly blood tests and limits patients’ dependence on hospitals, and the low cost of the drugs are valuable elements, even for countries with limited access to innovative or expensive drugs.

## Introduction

One of the emerging strategies to achieve disease control in advanced breast cancer while reducing the impact of toxicity is metronomic chemotherapy (mCT) [1]. mCT refers to the optimal biological dose, defined as the minimum biologically effective dose of a chemotherapeutic agent given as a continuous dosing regimen, with no prolonged drug-free breaks, that leads to anti-tumor activity [2].

A strong rationale supports the choice of a combination regimen when using a metronomic schedule: data from preclinical studies suggest that the metronomic combination of two different drugs allows the use of lower doses while still having an anti-tumor effect [3].

Several phase II studies have investigated metronomic vinorelbine (mVNR) in the treatment of breast cancer [4–8]. mVNR demonstrated long-lasting disease control combined with a good toxicity profile. Furthermore, a synergistic effect has been shown for VNR and capecitabine (CAPE), even when administered at standard schedules and doses [9, 10].

Our group recently published the results of the VICTOR-1 study, showing that the all-oral metronomic combination of VNR and CAPE is highly active in a population of hormone receptor (HR)-positive/HER2-negative advanced breast cancer patients, with a very low incidence of Grade 3–4 toxicity [4].

The phase II VICTOR-2 study was designed with the aim of confirming the results of the previous trial in a larger cohort of breast cancer patients.

## Patients and methods

VICTOR-2 is an open-label, phase II, multicenter trial conducted in 12 Italian centers between August 2011 and May 2015.

The study was conducted in accordance with the 1987 Declaration of Helsinki and adhered to Good Clinical Practice guidelines. Approval of the protocol was obtained from the local ethics committee for each participating center; all patients were required to give written informed consent before enrolment and to comply with the protocol for the duration of the study.

### Patients

Eligible patients were female, ≥18 years, with documented locally advanced, metastatic breast cancer, both previously treated or chemotherapy-naïve. Other inclusion criteria included HER2-negative disease (IHC 0-1 or IHC 2, confirmed as FISH negative), ≥1 measurable lesion according to RECIST 1.0 criteria and a life expectancy of ≥16 weeks. Previous endocrine therapy for advanced disease was allowed. Patients were required to have adequate bone marrow, hepatic, and renal functions, indicated by hemoglobin ≥10 g × 100 mL, absolute neutrophil count ≥2 × 10^9^/L, platelet count ≥100 × 10^9^/L, total serum bilirubin <1.5× upper normal limit (UNL), AST/ALT <2.5× UNL, (<3.5× UNL for liver metastases), and alkaline phosphatase <2.5× UNL (<5× UNL for bone metastases).

Patients were ineligible if they had only local relapse, previous exposure to a vinca alkaloid or CAPE, serious comorbidities such as cardiac disease, uncontrolled diabetes or hypercalcemia, severe peripheral neuropathy, active infection, or previous organ allograft. Patients were also excluded if they were pregnant or lactating; had clinical central nervous system or leptomeningeal metastases, a malabsorption disease, hypersensitivity to fluoropyrimidine therapy; had participated in another clinical trial with any investigational drug within 30 days before study inclusion; or had a history of another malignancy. Drugs acting on P450 cytochrome were not allowed during the study.

Patients were divided in two groups, according to treatment line (first-line = Group 1; ≥ second-line = Group 2).

### Treatment and dose modifications

Treatment consisted of VNR 40 mg each alternative day of the week (Monday, Wednesday, and Friday) and CAPE 500 mg three times a day (TID) after meals, given continuously without drug-free periods, until disease progression, unacceptable toxicity, or patient’s refusal. Patients’ compliance was evaluated by a diary given at the beginning of each cycle (1 cycle = 3 weeks). The dose of VNR was temporarily reduced to 30 mg three times a week at the first appearance of Grade 2 neutropenia or thrombocytopenia; the dose was increased to the previous level (40 mg) only if a complete recovery was observed at the beginning of the subsequent cycle. If a second episode of Grade 2 neutropenia or thrombocytopenia occurred, the dose was maintained at 30 mg until the end of the study with no further reduction. In the case of Grade 3–4 neutropenia or thrombocytopenia, VNR was interrupted for a maximum of 3 weeks, until recovery of neutrophil count at 1.0 × 10^9^/L; the dose administered upon resuming treatment was determined according to the toxicity grade. CAPE was reduced to 1000 mg/day in case of Grade 3–4 neutropenia or thrombocytopenia, or Grade 2–3 diarrhea or hand-foot syndrome, until recovery to Grade 1. For any other Grade 3–4 toxicity, both drugs were interrupted until recovery to lower grade.

### Outcomes

The primary endpoint of the study was CBR, defined as the proportion of patients with complete (CR) or partial response (PR) or with stable disease (SD) at 24 weeks from the start of treatment. Patients without a computed tomography (CT) re-evaluation at week 24 were considered non-responder if they discontinued treatment for medical decision, clinical progression, death, or toxicity.

Secondary endpoints were the objective response rate (ORR) and disease control rate (DCR), defined as the percentage of patients with CR + PR or CR + PR + SD, respectively, according to RECIST criteria. Further assessments included disease-free interval (DFI), progression-free survival (PFS), and time to progression (TTP). For patients achieving a CR or PR, the time to response and duration of response were also assessed.

### Assessments

Blood tests evaluating hepatic and renal function together with CEA and CA 15.3 were conducted at baseline and every 3 cycles, until study end. For each cycle white blood cells, erythrocyte count, hemoglobin, and platelets were assessed, before chemotherapy delivery. Tumor status was assessed according to RECIST 1.0 criteria, every 3 cycles (9 weeks) until disease progression, interruption of the treatment for toxicity, or patient’s refusal.

### Statistical methods

A sample size for each group has been defined according to the Fleming approach, modified by A’Hern [11]. We assumed the treatment had no therapeutic interest with a CBR ≤ 40 % for Group 1 and ≤20 % for Group 2, while a CBR ≥ 55 % and ≥35 %, respectively, was required to consider the treatment active. With a one-sided alpha level of 10 % and a power of 85 %, a total of 61 patients in Group 1 and 49 patients in Group 2 had to be enrolled. Considering a possible drop-out of about 10 %, 120 patients were required to have 105 patients evaluable for the primary endpoint (60 in Group 1 and 45 in Group 2).

CBR, ORR, and DCR were given as point estimate and 95 % confidence interval (CI). CIs were computed using exact binomial methods. Subjects who were not reported as having died or with progression/relapse at the time of the analysis were censored at their last available contact date. Survival data were summarized by median and interquartile range (IQR), computed with the Kaplan–Meier method. A Cox proportional hazard model was used to assess the impact of clinical factors on survival endpoints and results were expressed as hazard ratios (HR) and 95 % CI. Compliance with treatment and toxicity were evaluated using both cycle and patient as units of analysis. All analyses were conducted on the whole population and according to treatment line. Exploratory analyses were conducted on subgroups defined by HR status and metastatic site. Analyses were carried out with SAS (Version 9.2).

## Results

### Patient characteristics

**Fig. 1:**
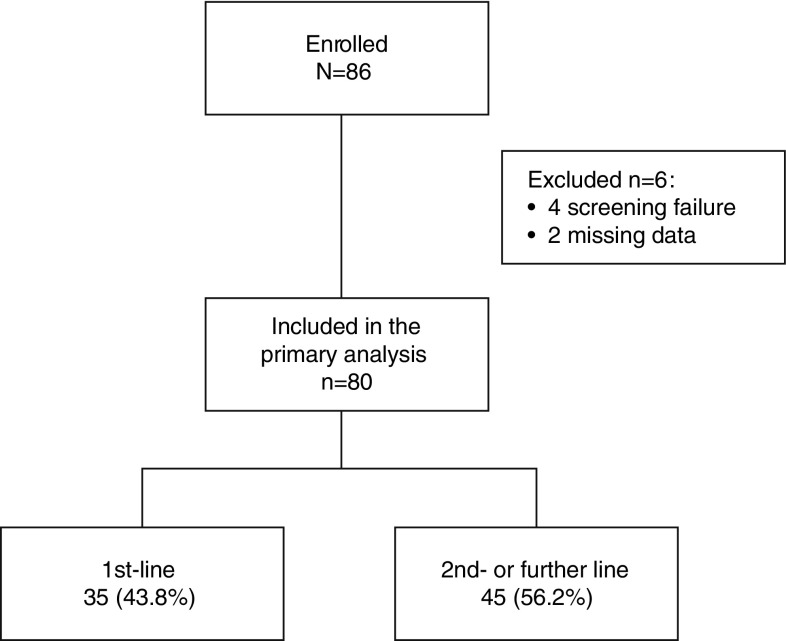
Study population

Between August 2011 and May 2015, 86 patients were enrolled. Six patients were subsequently excluded from the analysis, due to screening failure (*N* = 4) and missing data (*N* = 2) (Fig. 1). After the enrollment of 35 patients in Group 1 and 45 in Group 2, the study was prematurely closed, due to the slow recruitment of first-line patients. The minimum number of responding patients required to demonstrate clinical activity was reached in Group 2; thus enrollment was simultaneously closed in both groups. With 35 patients enrolled in Group 1, the power to test the original hypothesis is about 65 %; the purpose of analysis in this group, therefore, is merely descriptive.

The median age was 65.3 (56.0–69.3) years; most patients (65 %) had HR-positive disease (Table 1). Median DFI was 4.3 years (IQR 2.1–10.6). Sixty patients (76.9 %) had >2 metastatic sites and 58 (72.6 %) had visceral disease. At enrollment, 70 patients (87.5 %) had already received at least one prior antiblastic regimen; 35 patients (43.7 %) had been treated with anthracyclines, taxanes, or the combination.

**Table 1:** Patients and tumor characteristics

	First-line *N* = 35	Second-line *N* = 45	Overall *N* = 80
Number of patients *N* (%)	35 (43.8)	45 (56.2)	80.0
Age (years)			
Median (Q1–Q3)	66.3 (56.4–76.5)	64.9 (55.7–68.2)	65.3 (56.0–69.3)
Min–Max	38.0–85.6	44.0–82.7	38.0–85.6
Receptor status (*N*) %			
HR-positive	22 (62.9)	30 (66.7)	52 (65.0)
Triple-negative	13 (37.1)	15 (33.3)	28 (35.0)
Metastatic site *N* (%)			
Only bone with/without other site	9 (25.7)	8 (17.8)	17 (21.2)
Only visceral with/without other site	13 (37.1)	16 (35.6)	29 (36.3)
Visceral and bone with/without other site	10 (28.6)	19 (42.2)	29 (36.3)
Other site	3 (8.6)	2 (4.4)	5 (6.2)
Number of metastatic sites *N* (%)			
1	2 (6.1)	3 (6.7)	5 (6.4)
2	3 (9.1)	10 (22.2)	13 (16.7)
>2	28 (84.8)	32 (71.1)	60 (76.9)
Not reported	2	0	2
Chemotherapy for metastatic tumor* *N* (%)			
Yes	0 (0.0)	38 (86.4)	38 (54.3)
No	26 (100)	6 (13.6)	32 (45.7)
Metastatic treatment *N* (%)			
Only anthracyclines		1 (2.6)	1 (2.6)
Only taxanes		7 (18.4)	7 (18.4)
Only other		3 (7.9)	3 (7.9)
Anthracyclines and taxanes		8 (21.1)	8 (21.1)
Anthracyclines and other		2 (5.3)	2 (5.3)
Taxanes and other		12 (31.6)	12 (31.6)
Anthracyclines and taxanes and other		5 (13.2)	5 (13.2)

*N* total number of subjects, *Q1–Q3* first-third quartile, *Min–Max* minimum–maximum value

* Among the 70 patients that had already received at least one prior antiblastic regimen at study enrollment (either in the adjuvant and/or the metastatic setting)

### Treatments received

A total of 868 cycles were administered with a median of 9 (range 1–59) cycles per patient. A full dose of both drugs was administered for 76.5 % of cycles (89.2 and 67.8 % in Group 1 and 2, respectively). Forty-two patients (52.6 %) had dose reduction of the study drugs. At the final analysis, treatment had been discontinued in 75 patients for disease progression or death (*N* = 61, 81.4 %), toxicity (*N* = 8, 10.7 %), or physician decision (*N* = 5, 6.7 %).

### Efficacy

The CBR was 48.8 % (95 % CI 37.4–60.2) in the overall population, 45.7 % (95 % CI 28.8–63.4) in Group 1, and 51.1 % (95 % CI 35.8–66.3) in Group 2 (Table 2). Regarding receptor status, the CBR was 55.8 % (95 % CI 41.3–69.5) in HR-positive patients and 35.7 % (95 % CI 18.6–55.9) in triple-negative breast cancer (TNBC) patients; according to metastatic site the CBR was 59.1 % (95 % CI 36.4–79.3) in patients without visceral involvement and 44.8 % (95 % CI 31.7–58.5) in those with such involvement. The median duration of CB was 5.8 months (IQR 2.3–14.3) in Group 1 and 6.0 months (IQR 3.7–15.5) in Group 2.

**Table 2:** Clinical benefit rate according to pre-specified subgroups

*N*  total number of subjects, *95* *% CI* 95 % confidence interval, *HR* hormone receptor, *TNBC* triple-negative breast cancer

Patients in Group 1 had higher ORR (35.5 %, 95 % CI 19.2–54.6) and DCR (74.2, 95 % CI 55.4–88.1) than those in Group 2 (ORR 25.6, 95 % CI 13.5–41.2, DCR 67.4, 95 % CI 51.5–80.9) (Table 3). The median time to response was comparable in the two groups (2.1 months overall; IQR 2.1–4.1). The median duration of response was 11.3 and 6.4 months for Group 1 and Group 2, respectively.

**Table 3:** Objective response rate, disease control rate, duration of disease control, and time to response

Objective response rate (ORR)	First-line (Group 1)	Second-line (Group 2)	Overall
	*N* = 31	*N* = 43	*N* = 74
Responders (CR + PR): *n* (%)	11 (35.5)	11 (25.6)	22 (29.7)
[95 % CI]	[19.2–54.6]	[13.5–41.2]	[19.7–41.5]
Disease control rate (DCR)			
Responders (CR + PR + SD): *n* (%)	23 (74.2)	29 (67.4)	52 (70.3)
[95 % CI]	[55.4–88.1]	[51.5–80.9]	[58.5–80.3]

	First-line (Group 1)	Second-line (Group 2)	Overall
	*N* = 11	*N* = 11	*N* = 22
Kaplan–Meier estimate of duration of objective response (months)			
Median	11.3	6.4	8.2
(IQR)	4.1-not reached	5.3–12.8	5.2–12.8
Time to objective response (months)			
Median	2.1	2.1	2.1
(IQR)	2.1–5.0	2.1–3.4	2.1–4.1

*N* total number of subjects, *IQR* interquartile range, *95* *% CI* 95 % confidence interval, *CR* complete response, *PR* partial response, *SD* stable disease

The median TTP was 7.9 months (IQR 5.3–12.8) in Group 1 and 7.2 months (IQR 2.8–11.5) in Group 2. No difference in TTP was observed according to metastatic site or biological subtype (Table 4).

**Table 4:** Time to progression (TTP) in the whole population and according to hormone receptor status

		First-line	Second-line	Overall
		*N* = 35	*N* = 45	*N* = 80
Whole population				
Kaplan–Meier estimate of TTP (months)				
Median		7.9	7.2	7.5
(IQR)		5.3–12.8	2.8–11.5	3.7–11.5
According to HR status				
HR status				
TNBC * N* = 28	Progression/*N* (%)	7/13 (53.8)	15/15 (100)	22/28 (78.6)
	Kaplan–Meier estimate of TTP (months)			
	Median	7.2	4.3	6.5
	(IQR)	6.3–19.8	2.3–9.5	2.8–11.5
HR-positive * N* = 52	Progression/*N* (%)	18/22 (81.8)	25/30 (83.3)	43/52 (82.7)
	Kaplan–Meier estimate of median TTP (months)			
	Median	7.9	8.6	8.3
	(IQR)	5.3–11.3	3.3–13.9	4.6–12.8
Hazard ratio [95 % CI] (HR-positive vs TN)		1.30 [0.53–3.18]	0.73 [0.37–1.43]	0.89 [0.52–1.51]
*P* value		0.568	0.295	0.755

*N* total number of subjects, *IQR* Interquartile range, *HR* hormone receptor, *TNBC* triple-negative breast cancer, *TTP* time to progression, *95* *% CI* 95 % confidence interval

**Fig. 2:**
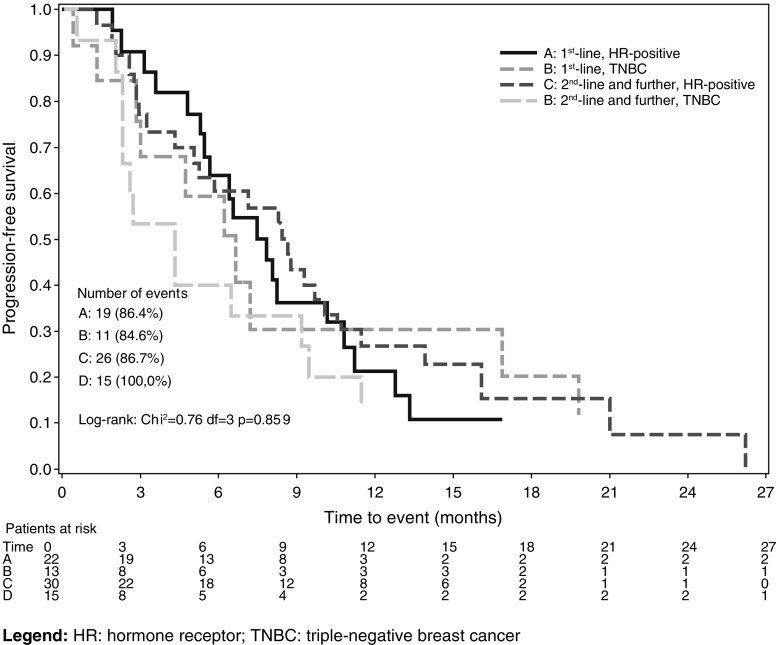
Progression-free survival according to treatment line and hormone receptor status

After a median follow-up of 18 months, 65 patients progressed, and 6 died. The median PFS was 6.7 months (IQR 4.74–11.3) in Group 1 and 7.2 months (95 % CI 2.8–11.5) in Group 2 while, according to biological type, the median PFS was 8.2 months in HR-positive patients and 4.7 months in TNBC patients (Fig. 2). PFS rate at 1 year according to the line of treatment was 24.3 and 22.2 % for Group 1 and 2, respectively.

### Treatment-related toxicities

The most frequent severe (Grade 3–5) toxicities in the 896 cycles delivered were non-febrile neutropenia (1.1 % of cycles), hand-foot syndrome (1.0 %), nausea/vomiting (1.0 %), leucopenia (0.8 %), fatigue (0.7 %), and diarrhea (0.4 %) (Table 5). No severe alopecia was observed.

**Table 5:** Percentages of G3 + G4 + G5 toxicity in treatment cycles. (Unit of analysis = cycle)

	First-line	Second-line	Overall
	*N* = 365	*N* = 531	*N* = 896
Non-febrile neutropenia	1.6	0.8	1.1
Hand and foot syndrome	0.5	1.3	1.0
Nausea and vomiting	0.8	1.2	1.0
Leucopenia	1.1	0.6	0.8
Fatigue	1.1	0.4	0.7
Diarrhea	0.5	0.4	0.4
Febrile neutropenia	0.5	0.4	0.4
Allergic reaction	0.8	0.0	0.3
Mucositis	0.5	0.0	0.2
Thrombocytopenia	0.0	0.4	0.2
Anemia	0.3	0.0	0.1
Fever/infection	0.0	0.0	0.0
Alopecia	0.0	0.0	0.0
Total	7.7	5.5	6.4

*N* total number of cycles

Severe hematologic toxicities per patient included Grade 3–4 leucopenia (*N* = 7 patients, 8.8 %), febrile neutropenia (*N* = 4, 5 %), Grade 3–4 thrombocytopenia (*N* = 2, 2.5 %; however, no bleeding occurred), and Grade 3 anemia (*N* = 1, 1.3 %). Among severe non-hematological toxicities, nausea/vomiting (10.0 %), hand-foot syndrome (10.0 %), fatigue (6.3 %), and diarrhea (5.0 %) were the most common.

Most of Grade 3–4 events occurred during the first 3 cycles, after which the probability of an adverse event per treatment cycle dropped to <1 %.

## Discussion

To our knowledge, the VICTOR-2 study is the first multicenter prospective trial testing the fully oral metronomic combination of VNR and CAPE in a population of advanced HER2-negative breast cancer patients with pre-specified analyses of efficacy and safety according to biological subtype, line of treatment, and site of metastatic disease.

The metronomic combination of continuous oral VNR and CAPE resulted in a promising CBR in pretreated patients (51.1 %), while results for first-line patients, due to premature recruitment closure, are inconclusive. ORR and particularly DCR are of relevant clinical interest due to the low incidence of serious adverse events.

Several phase II studies have tested the metronomic administration of oral anticancer drugs, reporting CBRs of 31–53 % and ORRs of 19–52 % [12–14]. Most of these studies had small sample sizes and were conducted in heavily pretreated breast cancer patients; in some cases the schedule could not be defined as metronomic, making comparison difficult.

More recent trials tested different and more active drugs, mainly VNR and CAPE, reporting CBRs of 77–80 % and ORRs of approximately 50 %. In a small, single-center study of 34 elderly patients with metastatic breast cancer treated with oral mVNR, ORR was 38 % and CBR was 68 % [15]. Most patients were receiving the treatment in the first-line setting and this may partially account for the high CBR. In another study [6], the combination of a low protracted dose of temozolomide, mVNR, and radiotherapy for newly diagnosed brain metastases from breast cancer resulted in CBR of 77 % and ORR of 52 %. Finally, the single-center phase I/II VICTOR-1 study [4] reported similar results to those shown by other studies with a clinical benefit rate (CBR) of 58.1 %. Taken together, these data and those from the present study suggest that mCT, when administered with highly active and synergistic drugs such as VNR and CAPE, is able to induce encouraging DCR.

The VICTOR-2 trial is the first study reporting data on the activity of metronomic VNR and CAPE in TNBC patients, a population for which there is a strong medical need for safe and active treatments. CBR in this population was 35.7 % and median duration of CB was 11.3 months. The median time to objective response was 2.1 months: this finding is of particular importance in the presence of aggressive disease, as it suggests that mCT could be an option even in this subset of patients, debunking the myth that it should be reserved for heavily pretreated patients, for whom no other therapeutic options are available.

The incidence of Grade 3–4 toxicity was very low with the metronomic combination of VNR and CAPE (6.4 % in 896 cycles). These results are in accordance with those from the other studies [4, 5] and are particularly important considering that half of the patients were pretreated with at least one line of CT and the majority (72.6 %) had visceral involvement.

The results of this study indicate that there are no drug-cumulative effects: the highest incidence of serious events was observed in cycles 1–3 and 4–6 and was followed by a significant decrease in severe toxicity during the subsequent cycles. The lack of drug accumulation over time, at least for VNR, has previously been demonstrated [16]. The availability of active and highly tolerated metronomic regimens, such as the VICTOR combination, may allow long-term therapy.

In this study, DCR was 74.2 % in Group 1 and 67.4 % in Group 2 and median duration of disease control was 7.6 months; these results suggest the metronomic VICTOR combination represents a feasible option to optimize the balance between efficacy and tolerability. Furthermore, the long-lasting treatment with mVNR and mCAPE could account for the high PFS reported in our study, with more than a quarter of the patients alive and free from progression after 12 months of mCT.

The ever-increasing published evidence on the use of mCT [17], may now contribute to outlining the profile of the patients who are likely to benefit from this option: HR-positive tumors, indolent disease, and bone metastases are all characteristics well represented in the metronomic studies and should be considered for patient’s selection. In addition, the results from this study support the use of the metronomic VICTOR combination in first-line.

This fully oral therapy does not require frequent blood testing, and the easy schedule of administration means that patients can remain at home for the whole duration of treatment. Positive clinical outcomes as first-line therapy, together with very low toxicity, mean that metronomic regimens could serve as a bridge to transition HR-positive patients from endocrine therapy to more aggressive CT regimens. Furthermore, lack of alopecia, severe nausea, and vomiting and the very low incidence of severe complications are an added value in the palliative setting.

## Conclusion

The results of the VICTOR-2 study have demonstrated the efficacy and safety of the metronomic combination of VNR and CAPE in an unselected group of patients with metastatic breast cancer, strongly suggesting that continuous administration of low-dose drugs allows prolonged duration of treatment and positive clinical outcomes, while minimizing the risk of adverse events.

## Appendix

### **List of co-authors belonging to the VICTOR Study Group**

Francesca Riva, Monza, Italy; Davide Pelizzoni, Monza, Italy; Isabella Marchi, Modena, Italy; Elena Collovà, Legnano, Italy; Giuseppe Prati, Guastalla, Italy; Antonio Ardizzoia, Lecco, Italy; Davide Toniolo, Rho, Italy; Palma Pugliese, Como, Italy; Claudia Pogliani, Saronno, Italy; Abbondanza Gambino, Lecce, Italy; Lucia Stocchi, Cattolica, Italy; Andrea Colombo, Busto Arsizio, Italy; Cinzia Fasola, Milano, Italy; Raffaele Venezia, Gorgonzola, Italy; Fabio Galli, Milano, Italy; Valter Torri, Milano, Italy.
